# Mitochondria and the eye—manifestations of mitochondrial diseases and their management

**DOI:** 10.1038/s41433-023-02523-x

**Published:** 2023-04-25

**Authors:** Benson S. Chen, Joshua P. Harvey, Michael J. Gilhooley, Neringa Jurkute, Patrick Yu-Wai-Man

**Affiliations:** 1 John van Geest Centre for Brain Repair and MRC Mitochondrial Biology Unit, Department of Clinical Neurosciences, University of Cambridge, Cambridge, UK; 2 Cambridge Eye Unit, Addenbrooke’s Hospital, Cambridge University Hospitals, Cambridge, UK; 3 Moorfields Eye Hospital NHS Foundation Trust, London, UK; 4 Institute of Ophthalmology, University College London, London, UK; 5 The National Hospital for Neurology and Neurosurgery, Queen Square, University College London Hospitals NHS Foundation Trust, London, UK

**Keywords:** Clinical genetics, Eye manifestations, Therapeutics, Neurological disorders, Hereditary eye disease

## Abstract

Historically, distinct mitochondrial syndromes were recognised clinically by their ocular features. Due to their predilection for metabolically active tissue, mitochondrial diseases frequently involve the eye, resulting in a range of ophthalmic manifestations including progressive external ophthalmoplegia, retinopathy and optic neuropathy, as well as deficiencies of the retrochiasmal visual pathway. With the wider availability of genetic testing in clinical practice, it is now recognised that genotype-phenotype correlations in mitochondrial diseases can be imprecise: many classic syndromes can be associated with multiple genes and genetic variants, and the same genetic variant can have multiple clinical presentations, including subclinical ophthalmic manifestations in individuals who are otherwise asymptomatic. Previously considered rare diseases with no effective treatments, considerable progress has been made in our understanding of mitochondrial diseases with new therapies emerging, in particular, gene therapy for inherited optic neuropathies.

## Abstract (zh)

从历史上来看, 不同的线粒体疾病的症状在临床上可由眼科表现识别。由于线粒体与代谢活跃组织相关, 疾病常累及眼部, 导致一系列的眼部表现, 包括进行性眼外肌麻痹、视网膜视神经病变, 以及视交叉后路缺陷。随着基因检测在临床实践中的广泛应用, 研究人员已意识到线粒体疾病中的基因型-表型相关性可能不精确: 很多典型症状可能与多个基因和基因突变相关, 并且相同的基因突变可能有多种临床表现, 包括无症状者的亚临床表现。线粒体疾病之前认为是无有效治疗方法的罕见病, 随着新治疗方法的出现, 我们对线粒体疾病的理解取得了较大进步, 尤其表现在遗传性视神经病变的基因治疗。

## Introduction

**Fig. 1:**
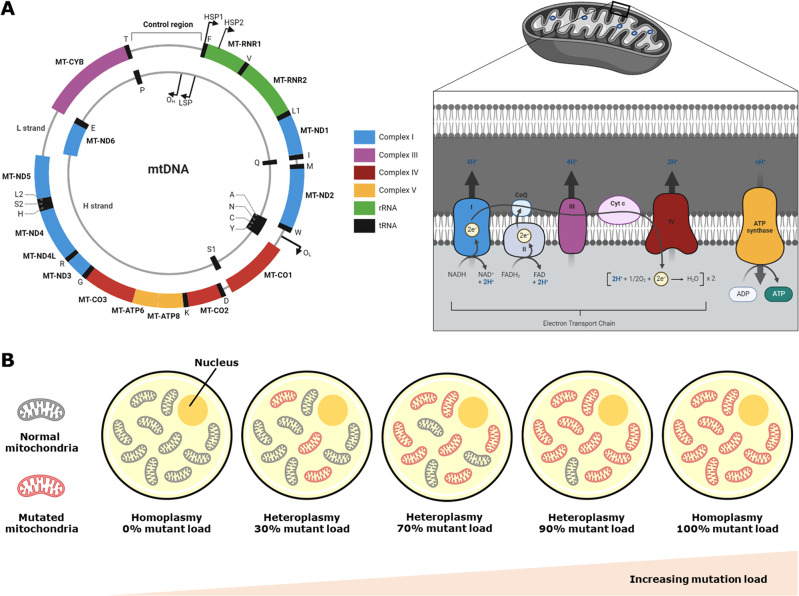
Overview of mitochondrial DNA (mtDNA) and heteroplasmy. **A**. The mitochondrial genome encodes 13 protein subunits, two ribosomal RNAs (rRNAs), and 22 transfer RNAs (tRNAs), organised in a circular structure. The 13 protein subunits instruct the cell to produce the protein subunits of the enzyme complexes of the mitochondrial respiratory chain, which is comprised of five enzyme complexes (I-V) and two mobile electron carriers, coenzyme Q10 (CoQ) and cytochrome c (Cyt c). mtDNA variants in genes encoding the protein subunits of the mitochondrial respiratory chain, may cause biochemical abnormalities that interfere with oxidative phosphorylation, thereby precipitating a bioenergetic defect and mitochondrial failure. Nuclear-encoded proteins (not depicted) also play an integral role in the replication, maintenance, transcription, and translation of mtDNA and mtDNA-encoded proteins. Variants in these genes can result in disturbed mtDNA integrity; defects in mtDNA replication and maintenance; defects in mitochondrial fusion and fission; and defects in nucleotide synthesis and salvage. **B**. Each human cell contains thousands of copies of mtDNA and they are usually all identical (homoplasmy). Individuals with mitochondrial diseases resulting from mutations in the mtDNA may harbour a mixture of normal and mutated mtDNA within each cell (heteroplasmy). As mutant load increases, the bioenergetic defect caused by biochemical abnormalities of mitochondrial respiration becomes increasingly severe, leading to a continuum of clinical presentations. Panel A adapted from ‘Human mtDNA Sequence Map’ and ‘Electron Transport Chain’, by BioRender.com (2022). Retrieved from https://app.biorender.com/biorender-templates (accessed 15 January 2022).

Mitochondrial diseases are a clinically heterogeneous group of disorders that arise as a result of dysfunction of the mitochondrial respiratory chain [1]. Historically, these diseases were classified into distinct syndromes based on their clinical presentations. However, with developments in whole genome sequencing technology and increased availability of genetic testing in the past decade, in particular mitochondrial DNA (mtDNA) sequencing, it is now recognised that many of these classic syndromes are associated with multiple genes and genetic variants, including variants in the nuclear DNA (nDNA) (Table 1). Additionally, the same genetic variant may have different clinical presentations depending on the level of tissue mutation load, a unique characteristic of mitochondrial disease. Within a cell, pathogenic mtDNA mutations are frequently mixed with normal mtDNA, a state known as heteroplasmy (Fig. 1). As the percentage of mutant mtDNA (mutant load) increases, the bioenergetic defect becomes increasingly severe, leading to a continuum of clinical presentations [1]. In this review, we provide an update on the ophthalmic presentations of mitochondrial disease and review their management, including extraocular manifestations, retinopathy, optic neuropathy, and visual dysfunction arising from dysfunction of the retrochiasmal visual pathways.

**Table 1:** Summary of classic mitochondrial diseases with ophthalmic manifestations.

Syndrome	Acronym	Genetic Variant	Clinical Features
Chronic Progressive External Ophthalmoplegia	CPEO	• Sporadic CPEO • **Single large** **de novo** **mtDNA deletions** (ranging from 1.3 to 9.5 kb); deletions ranging from 1.2 to 8 kb are associated with KSS and Pearson syndrome, whereas deletions from 2.3 to 9.5 kDA – with CPEO. Larger deletions associated with more severe disease. mtDNA duplications also identified in KSS and Pearson phenotypes Inherited CPEO • Autosomal dominant: ***POLG1***, *POLG2*, *ANT1*, *C10orf2 (twinkle)*, *RRM2B*, *DNA2*, *OPA1* • Autosomal recessive: *TYMP*, *POLG1*, *DGUOK*, *TK2*, *MGM1*, and *RNASEH1* • Maternal: *MT-TL1*, *MT-TQ*, *MT-TA*, *MT-TY*, *MT-TK*, *MT-TN*, *MT-TI*, *MT-TP*	CPEO, KSS, PS: Bilateral ptosis; diffuse ophthalmoplegia; pigmentary retinopathy in some cases of CPEO KSS: CPEO onset <20 years; pigmentary retinopathy; CSF protein >1 g/L, cerebellar ataxia; heart block; sensorineural hearing loss; myopathy, dysphagia; diabetes; endocrine dysfunction PS: Sideroblastic anaemia; pancytopaenia; exocrine pancreatic failure; renal tubular defects CPEO-plus: May have features of KSS or PS above; other manifestations include ataxia, Parkinsonism, seizures; tremor, peripheral neuropathy; gastrointestinal dysmotility
Kearns-Sayre Syndrome	KSS		
Pearson Syndrome	PS		
Neuropathy, Ataxia, Retinitis Pigmentosa	NARP	***MT-ATP6*** (m.8993T>G; m.8993T>C; m.8989G>C; m.8729G>A)	Late-childhood or adult-onset peripheral neuropathy; ataxia; pigmentary retinopathy (progressing to retinitis pigmentosa); basal ganglia lucencies; sensorimotor neuropathy
Maternally Inherited Leigh Syndrome	MILS	***MT-ATP6*** (m.8993T>G with heteroplasmy levels above 90%); *MT-ND3*; *MT-ND5*; *MT-ND6*	Subacute relapsing encephalopathy; cerebellar and brainstem signs; infantile onset; raised lactate concentration in blood or CSF; bilateral white matter lesions on neuro-imaging; pigmentary retinopathy
Maternally Inherited Diabetes and Deafness	MIDD	***MT-TL1*** (m.3243A>G)	Diabetes mellitus; bilateral sensorineural deafness; pattern dystrophy; short stature; cardiac abnormalities; myopathy; proteinuria; gastrointestinal disease
Mitochondrial Encephalomyopathy, Lactic Acidosis and Stroke-Like Episodes	MELAS	***MT-TL1*** (m.3243A>G with heteroplasmy levels of 50–90%)	Stroke-like episodes at age <40 years; seizures and/or dementia; ragged-red fibres and/or lactic acidosis; pigmentary retinopathy (variable degree), diabetes mellitus; cardiomyopathy; bilateral sensorineural deafness; cerebellar ataxia; optic atrophy; cataracts
Leber Hereditary Optic Neuropathy	LHON	***MT-ND4*** (m.11778G>A); ***MT-ND1*** (m.3460G>A); ***MT-ND6*** (m.14484T>C); *MT-ND2*; *MT-ND3*; *MT-ND4L*; *MT-ND5*; *MT-ATP6*; *MT-CO3*; *MT-CYB* *NDUFS2*, *NDUFAF5*, *DNAJC30*, *MCAT*	Acute-subacute bilateral sequential visual failure due to optic neuropathy; optic atrophy LHON-plus: May have features including myopathy; ataxia; peripheral neuropathy; movement disorder; multiple sclerosis; cardiac abnormalities; endocrine dysfunction; hearing loss; kidney disease
Dominant Optic Atrophy	DOA	***OPA1***	Slow progressive bilateral, relatively symmetric, visual failure due to optic neuropathy; optic atrophy DOA-plus: May have features including myopathy; ataxia; peripheral neuropathy; sensorineural hearing loss
Myoclonic Epilepsy and Ragged Red Fibres	MERRF	***MT-TK*** (m.8344A>G, m.8356T>C; m.8363G>A; m.8361G>A); *MT-TF*; *MT-TH*; *MT-TI*; *MT-TL1*; *MT-TP*; *MT-TS1*; *MT-TS2*	Myoclonus; seizures; cerebellar ataxia; myopathy; raised lactate concentration in blood or CSF; ragged red fibres on muscle biopsy; dementia; optic atrophy; bilateral deafness; peripheral neuropathy; spasticity; multiple lipomata; pigmentary retinopathy; ophthalmoplegia; cardiomyopathy
Mitochondrial Neurogastrointestinal Encephalopathy	MNGIE	***TYMP*** (biallelic pathogenic variants); *LIG3* (biallelic pathogenic variants)	Severe gastrointestinal dysmotility; cachexia; ptosis; ophthalmoplegia; sensorimotor neuropathy; diffusely abnormal white matter on neuro-imaging
Kjellin syndrome	SPG15	***ZFYVE26*** (biallelic pathogenic variants)	Pigmentary maculopathy in some individuals
*ACO2*-associated disease	OPA9	***ACO2*** (biallelic pathogenic variants)	Retinal degeneration, optic atrophy
*RTN4IP1*-associated disease	OPA10	***RTN4IP1*** (biallelic pathogenic variants)	Optic atrophy, mild retinal degeneration in some individuals
*SSBP1*-associated disease	OPA3	***SSBP1*** (monoallelic and biallelic pathogenic variants)	Optic atrophy, variable degree retinal dystrophy and foveopathy
*FDXR*-associated disease		***FDXR*** (biallelic pathogenic variants)	Variable pigmentary retinopathy, optic atrophy
Primary Coenzyme Q10 deficiency		Coenzyme Q10 biosynthesis pathway genes biallelic defects	Retinitis pigmentosa
*POLG*-associated disease		***POLG***	Pigmentary retinopathy
Pigmentary retinopathy in severe neurological phenotype		*MT-TE* variants (m.14685G>A, m.14710G>A)	Pigmentary retinopathy; ptosis; ophthalmoplegia

Genes frequently associated with mitochondrial syndromes are in bold. Primary clinical features are underlined.

*CSF* cerebrospinal fluid.

## Extraocular manifestations

**Fig. 2:**
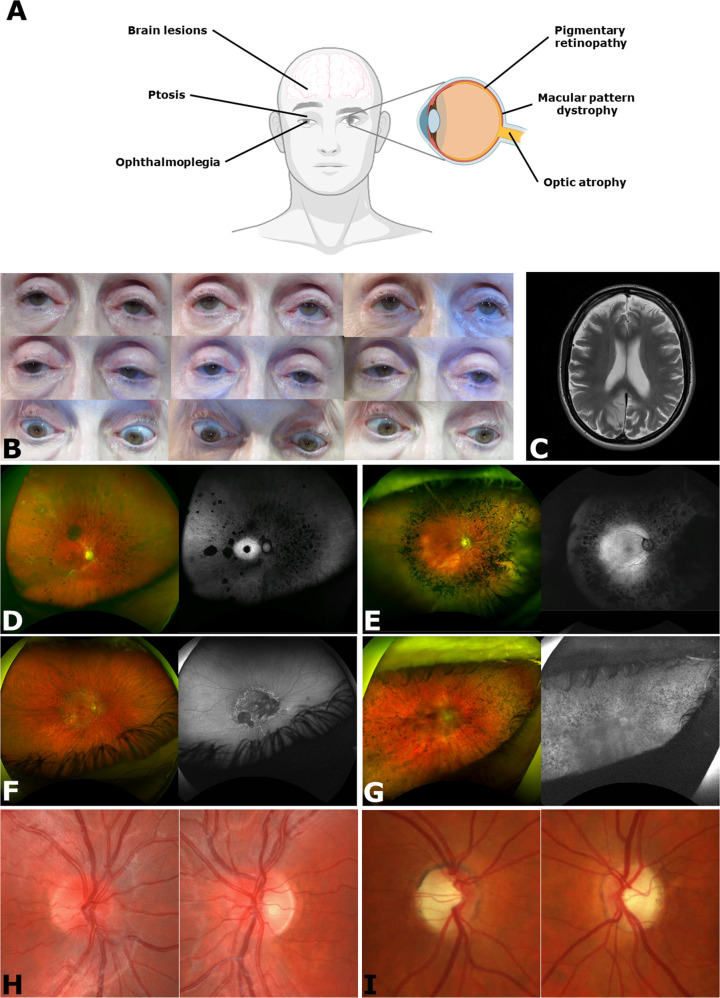
Overview of the ophthalmic manifestations of mitochondrial disease. **A** Mitochondrial dysfunction can affect many parts of the eye, resulting in multiple ophthalmic manifestations, including abnormalities of the cornea, lens, ciliary body, retina, and optic nerve. However, classic presentations of primary mitochondrial diseases typically affect: (1) extraocular muscles, resulting in ptosis and ophthalmoplegia; (2) outer retina, resulting in retinal pigmentary changes or macular pattern dystrophy; (3) inner retina, resulting in loss of retinal ganglion cells and optic atrophy; and (4) cerebral cortex and/or white matter, resulting in visual field defects and disorders of higher visual processing. **B** Features of CPEO including bilateral ptosis due to reduced levator palpebrae superioris function and diffuse ophthalmoparesis. **C** Axial T2-weighted MRI brain of a patient with MELAS due to the m.3243A>G mutation in *MT-TL1* and left homonymous hemianopia, demonstrating a discrete right medial occipital cortical lesion with associated oedema. Optos wide-field fundus imaging (pseudocolour imaging on the left and fundus autofluorescence on the right) of individuals (right eye only) carrying pathogenic *SSBP1* variants (**D**), *PDSS1* (**E**), *MT-TL1* (**F**), and *MT-ATP6* (**G**) variants. Pseudocolor imaging demonstrates various degrees of retinal vessels attenuation, pigmentary changes mainly localised in the mid-peripheral retina in *SSBP1-* and *PDSS1*-associated retinopathy and widespread pigmentary changes in *MT-ATP6-*associated retinopathy with various degrees of retinal atrophy observed in all affected individuals. Characteristic features of foveopathy is observed in *SSBP1* case; retinitis pigmentosa features with retinal vessels attenuation, pigmentary (bone spicule) changes in mid-periphery are seen in *PDSS1* case; and pattern dystrophy in *MIDD* case. Fundus autofluorescence image indicates areas of retinal atrophy with patches of decreased autofluorescence and demonstrates hypoautofluorescence corresponding to mid-peripheral pigmentary changes with a hyperautofluorescence ring at the macula delineating the border between normal and abnormal retina. **H** Fundus photographs of a patient with LHON due to the m.11778G>A mutation in *MT-ND4* experiencing acute onset vision loss in the left eye initially, followed 2 months later by vision loss in the right eye. The right optic nerve appears oedematous with hyperaemia and peripapillary telangiectasia. The left optic nerve appears pale temporally with resolving disc oedema. **I** Fundus photographs of a patient with DOA due to a pathogenic variant in *OPA1*. Both optic discs show temporal pallor and OCT (not shown) demonstrated diffuse loss of peripapillary RNFL with some nasal sparing and diffuse thinning of the GC-IPL. CPEO: chronic progressive external ophthalmoplegia; DOA: dominant optic atrophy; GC-IPL: ganglion cell-inner plexiform layer; LHON: Leber hereditary optic neuropathy; MELAS: mitochondrial encephalomyopathy lactic acidosis and stroke-like episodes; MRI: magnetic resonance imaging; OCT: optical coherence tomography; RNFL: retinal nerve fibre layer. **A** created with BioRender.com. **B** adapted from Jain, S. *Simplifying Strabismus: A Practical Approach to Diagnosis and Management*. Cham: Springer International Publishing; 2019. Images **D**–**G** courtesy of Professor Andrew R. Webster and Dr. Neringa Jurkute (Moorfields Eye Hospital NHS Foundation Trust, London, United Kingdom).

Given the high energy demand of muscle tissue, extraocular muscle dysfunction is one of the major clinical manifestations of mitochondrial disease. First described by Von Graefe in 1868 [2], the syndrome of progressive external ophthalmoplegia (often termed ‘chronic progressive external ophthalmoplegia’ or CPEO) is a constellation of clinical findings relating to myopathy of the extraocular muscles, including bilateral ptosis and symmetrical reduction in ocular movements due to myopathy and, in later stages, to fibrotic restriction (Fig. 2). Affecting ~3.4 people per 100,000, CPEO can present on a spectrum between an isolated CPEO phenotype and disorders where CPEO is one feature of a multisystem syndrome—as a group termed CPEO ‘plus’ (Table 1) [3].

The onset of CPEO is often insidious and while it can occur at any age, it is often seen in young adults (mean age 29 years) presenting with bilateral symmetrical ptosis as the result of poor function of the levator palpebrae superioris [3–5]. Classically, diplopia was not considered a presenting feature: the symmetrical nature of the extraocular myopathy and the ptosis itself (and in many patients a degree of visual suppression) preventing diplopia in the early stages. However, several case series have reported diplopia in up to 50% of cases, and early oculomotor changes can often be seen on examination including slowed saccades before any deviation becomes manifest [6, 7].

### Clinical spectrum of Chronic Progressive External Ophthalmoplegia (CPEO)

Several syndromes include a CPEO presentation (Table 1)—perhaps the most notable CPEO plus syndrome was described by Kearns and Sayre [8], in which CPEO is accompanied by pigmentary retinopathy (discussed in Retinal Manifestations below), cardiac conduction defects, raised cerebrospinal fluid protein levels, and cerebellar ataxia [9]. With a prevalence of ~1.5 per 100,000 in one study [10], the Kearns Sayre Syndrome (KSS) generally presents in young adults and represents an intermediate phenotype along the spectrum of disease severity. At the more severe end of this spectrum lies the Pearson Syndrome, where large-scale mitochondrial DNA deletions lead to respiratory chain dysfunction manifesting with sideroblastic anaemia, exocrine pancreatic dysfunction, and myopathy among other features usually leading to death in infancy [11, 12]. In patients with the Pearson Syndrome who survive infancy, CPEO often develops together with pigmentary retinopathy and Leigh-like Syndrome (discussed in ‘Retinal Manifestations’ below) in early life.

This spectrum of severity is reflected in the genetic heterogeneity underlying CPEO. While around half of solved cases are sporadic, 50% are inherited either in an autosomal fashion or maternally (Table 1) [7]. Like many mitochondrial disorders, there can be great variability in the phenotypes of patients with the same genetic diagnoses, which are thought to relate to the level of mitochondrial heteroplasmy or exposure to environmental mitochondrial toxins [13, 14].

#### Extraocular manifestations—management

Whilst the clinical diagnosis is often not in doubt, several conditions (Table 2) can present with some features common to CPEO and these should be excluded by appropriate examination or investigation, especially if genetic testing for a genetic variant associated with CPEO is uninformative. Additionally, all patients with CPEO should undergo a dilated fundus examination to visualise retinal abnormalities that might suggest KSS. If KSS is suspected clinically, neurological assessment and additional investigations such as cardiac assessment and lumbar puncture should be considered.

**Table 2:** Differential diagnoses of Chronic Progressive External Ophthalmoplegia.

•Myasthenia gravis
• Medications: antiretrovirals; statins
• Sagging eye syndrome
• Wernicke encephalopathy
• Thyroid eye disease
• Progressive supranuclear palsy
• Miller Fisher syndrome
• Congenital cranial dysinnervation disorders: congenital fibrosis of the extraocular muscles (CFEOM); Moebius syndrome; Duane syndrome etc

### Management of ptosis

Ptosis associated with CPEO can be challenging both for the patient (who may adopt an altered head position, have their visual axis obscured or be troubled by poor cosmesis) and the surgeon (with continued progression, absent Bell’s reflex and poor residual levator palpebrae superioris function (LPF)). In early disease, where there is residual LPF, surgical approaches to augment this muscle can be useful [15]. However, as muscle function becomes impaired with progression of the disease, suspensory approaches with fascia lata or, more often, silicone are indicated [4, 16, 17]. Conservative measures such as ptosis props and scleral contact lenses have a role and, while they may be poorly tolerated early in disease, they may have an adjunctive role when surgical options have been exhausted or are not appropriate [15].

### Management of ophthalmoplegia

Symptomatic ophthalmoplegia is managed with a combination of conservative approaches, botulinum toxin, and surgical interventions. The progressive, symmetrical nature of ophthalmoplegia may necessitate a nuanced discussion with patients regarding the need for repeated interventions over their lifetime and patients should be carefully consented for the high likelihood of multiple procedures, and the need for adjuncts as the disease progresses.

While occlusion will resolve diplopia, Fresnel prisms can be useful in managing a varying deviation as they can be easily adjusted over time. Prisms can also be helpful for small residual angles, particularly as there is suggestion that CPEO patients can have narrower fusional amplitudes [18]. Botulinum toxin alone, although insufficient for the larger exodeviations seen in CPEO, may be particularly useful for residual angles that may develop post-surgically [19, 20].

Strabismus surgery for CPEO should be carefully planned and patients informed that the deviation will invariably return given the progressive nature of the underlying cytopathy. Forced duction testing can be very useful, as under-action and failure of ipsilateral antagonist muscles to relax can both lead to deviations. It has been suggested that muscle resection can be more effective than recession [21]. However, one larger case series has suggested near-maximal horizontal surgery using adjustable sutures to be the most effective [20].

## Retinal manifestations

Although primary retinal ganglion cell (RGC) loss involving the inner retina and optic atrophy are classical manifestations of mitochondrial disease (discussed in ‘Optic Neuropathy’ below), outer retinal involvement is also observed as part of the phenotype of several syndromic central nervous system diseases caused by mtDNA mutations. Additionally, pathogenic defects in nDNA have also been identified to cause combined optic atrophy and retinal dystrophy [22]. In the outer retina, the photoreceptors, retinal pigment epithelium (RPE), and Müller cells contain large numbers of mitochondria, making these cell populations vulnerable to mitochondria dysfunction and oxidative damage [23]. The retina can be involved as part of the phenotype of several mitochondrial syndromes including KSS; Neuropathy, Ataxia, Retinitis Pigmentosa (NARP); Maternally Inherited Leigh Syndrome (MILS); and Maternally Inherited Diabetes and Deafness (MIDD) (discussed in more detail below) [22]. Historically, various terms have been used to describe the retinal manifestations of mitochondrial disease, including ‘pigmentary degeneration’ and ‘salt and pepper retinopathy’ [9, 24]. Identification of these classic mitochondrial retinopathies may help facilitate the clinical and molecular diagnosis of specific mitochondrial diseases.

A recent classification system, based on multimodal imaging findings from 23 patients with retinopathy and genetically-defined mitochondrial disease, identified three distinct phenotypes of mitochondrial retinopathy [25]. Sporadic single large-scale mtDNA deletions, such as those associated with CPEO, exhibited either mild, focal pigmentary abnormalities on ophthalmoscopy (Type 1 mitochondrial retinopathy; usually visually asymptomatic), or widespread granular fundus alterations on ophthalmoscopy and autofluorescence (Type 3 mitochondrial retinopathy; frequently associated with nyctalopia and severe widespread visual dysfunction). Type 2 mitochondrial retinopathy was usually associated with the m.3243A>G mutation in *MT-TL1* (the most common disease-causing mtDNA mutation with a carrier rate of ~1 in 400 people) [26] and displayed a spectrum of manifestations including multifocal white-yellowish subretinal deposits and pigmentary changes (limited to the posterior pole) or chorioretinal atrophy with or without foveal involvement. Patients frequently reported problems in dim light and, if they had chorioretinal atrophy, severe central vision loss.

### Neuropathy, Ataxia, Retinitis Pigmentosa (NARP) and Maternally Inherited Leigh Syndrome (MILS)

NARP is a progressive neurodegenerative disease that presents clinically with the features in its name and other associated findings, including seizures, cognitive impairment, and developmental delay [27]. The characteristic ocular finding is a salt and pepper retinopathy that appears early in the disease course and eventually progresses to retinitis pigmentosa [28]. Other visual symptoms include nyctalopia and constriction of visual fields.

NARP is primarily caused by the m.8993T>G/C mutation in *MT-ATP6*, leading to complex V dysfunction and impaired ATP production [27, 29]. This particular mtDNA variant is also the most common one associated with MILS, a severe multisystem mitochondrial disorder characterised by encephalopathy, lactic acidosis, cardiomyopathy, and respiratory dysfunction [30]. In contrast to NARP, which generally presents in childhood, the first signs of MILS are seen in infancy [31]. The heteroplasmy level of the mutant mtDNA species is helpful in determining the phenotypic expression of the mtDNA variant, with a high mutation load (>90%) causing the more severe MILS phenotype and a mutation load 60–90% causing NARP [32, 33].

### Maternally Inherited Diabetes and Deafness (MIDD)

First described by Van den Ouweland et al. [34], MIDD is a multisystem disorder characterised by maternally transmitted diabetes mellitus and sensorineural hearing deafness. Most cases of MIDD are due to the aforementioned m.3243A>G mutation in *MT-TL1*, encoding a mitochondrial transfer RNA. It should be noted that the m.3243A>G mutation is clinically heterogeneous and, depending on mutant load and tissue level, can present as MIDD; Mitochondrial Encephalomyopathy Lactic Acidosis and Stroke-like episodes (MELAS); CPEO; an overlap syndrome; or with a clinical phenotype that does not fall within the criteria for currently recognised mitochondrial syndromes [26].

Most patients with MIDD remain visually asymptomatic with good visual acuity. Despite diabetes mellitus being a defining feature, the prevalence of diabetic retinopathy is lower than those with type 2 diabetes mellitus of similar duration [35, 36]. Instead, the majority of patients with MIDD exhibit a specific macular pattern dystrophy (mean age at detection 46.5 years, range 27–71 years) [35, 36], characterised by RPE hyperpigmentation that surrounds the macula or is more extensive and encompasses the optic disc [35, 37, 38]. A unique feature of the macular pattern dystrophy in MIDD, that may help to distinguish it from other maculopathies, is the finding of occult RPE disruption seen on autofluorescence imaging as a diffuse speckled pattern, extending beyond the macular abnormalities seen on ophthalmoscopy [38, 39]. Autofluorescence imaging has been suggested as a useful marker of disease progression [40, 41]. As the disease progresses, areas of speckled pigments on autofluorescence evolve into areas of atrophy, with relative sparing of the fovea until the advanced stages of the disease.

Multimodal imaging, in particular, OCT and wide-field autofluorescence, should be performed as subtle outer retinal changes of mitochondrial retinopathies may not be obvious on ophthalmoscopy. Visual electrophysiology testing including visually-evoked potentials, pattern electroretinogram and full-field electroretinogram should also be considered, especially in subclinical forms of mitochondrial retinopathies as they may help to localise the abnormality and detect outer retinal dysfunction while there are no structural changes observed.

#### Retinal manifestations—management

Although genotype-phenotype correlations are not always clear-cut, the specific pattern of retinal abnormalities may be helpful in guiding genetic testing and subsequent management. Macular pattern dystrophy in association with hearing loss are defining features of MIDD and this combination should prompt testing for the m.3243A>G mutation in *MT-TL1*. As the m.3243A>G mutation is associated with both MIDD and MELAS, patients with this mutation should be referred for multidisciplinary investigations to identify other organ involvement, including sensorineural hearing loss, diabetes, cardiac conduction defects and kidney disease.

There are currently no specific pharmacological treatments for mitochondrial retinopathies. Those with advanced disease involving the fovea or with chorioretinal atrophy may benefit from supportive management, such as visual impairment registration and referral to a low vision clinic.

## Optic neuropathies

Inherited optic neuropathies (IONs) are characterised by bilateral and progressive degeneration of the optic nerve secondary to a genetic variant which invariably affects mitochondrial function [42]. Clinically, they are characterised by loss of visual acuity/field and colour vision caused by RGC loss. The archetypical ION is an isolated bilateral optic neuropathy as seen in Leber hereditary optic neuropathy (LHON) and dominant optic atrophy (DOA). However, many IONs can present as part of a systemic syndrome such as Wolfram syndrome, where the optic atrophy is accompanied by diabetes insipidus, diabetes mellitus and sensorineural hearing loss. Furthermore, a subgroup of LHON and DOA can sometimes present with extraocular features referred to as LHON “plus” and DOA “plus”, respectively. It should be stressed, however, that establishing causation between these extraocular features and the underlying mtDNA or nDNA genetic variant is not always straightforward.

### Leber Hereditary Optic Neuropathy (LHON)

Considered the prototype of mitochondrial disease, LHON is an important cause of inherited mitochondrial blindness, affecting ~ 1 in 30,000 individuals [43]. In most affected individuals (peak age 15–35 years), painless bilateral sequential vision loss is the only symptom of LHON, with an inter-eye delay of weeks to months. Visual acuity declines rapidly to 20/200 or less, reaching a nadir ~ 4–6 weeks after onset. Long-term visual prognosis is generally poor for most patients with visual acuity worse than 20/400 [44].

Three primary mtDNA point mutations (m.3460G>A in *MT-ND1*, m.11778G>A in *MT-ND4* and m.14484T>C in *MT-ND6*) account for ∼ 90% of LHON cases globally [42]. These three mutations all involve genes encoding subunits of complex I, the first enzyme of the mitochondrial respiratory chain. In LHON, defective mitochondrial oxidative phosphorylation precipitates a bioenergetic crisis and elevated levels of reactive oxygen species (ROS), leading to RGC dysfunction and release of signalling factors that trigger cellular apoptosis [23]. The m.14484T>C mtDNA variant and onset of LHON before the age of 12 (childhood LHON) are associated with a relatively more favourable visual prognosis [45].

Not all individuals who carry a genetic mutation associated with LHON will experience visual loss. The penetrance of LHON has often be reported as being ~50% among male carriers and ~10% among female carriers, highlighting the marked sex bias. However, in a recent national Australian cohort, the penetrance of LHON was reported to be lower at 17.5% for male carriers and 5.4% for female carriers [46]. These differences could reflect other genetic and environmental influences with the latter possibly having changed over time e.g. smoking habits. This incomplete penetrance is not fully explained by mitochondrial heteroplasmy, as most LHON carriers are homoplasmic. External metabolic stressors, such as smoking, excessive alcohol consumption and certain toxins, have been identified as contributory factors leading to disease conversion [47]. Hormonal influences are also thought to modulate the risk of visual loss with oestrogens being protective for female LHON carriers [48, 49].

### Dominant Optic Atrophy (DOA)

DOA is the most common ION with an estimated minimum prevalence of 1 in 25,000 [50]. Visual loss typically presents in the first two decades of life with the majority of patients declining steadily over time to reach the criteria for registration as visually impaired. *OPA1* (3q21) is thought to be the causative gene in over 60–70% of DOA cases [51]. Over 450 pathogenic variants of *OPA1* have been described with missense variants and variants located in the GTPase/dynamin domain associated with a worse ocular phenotype and a higher probability of DOA ‘plus’ [52, 53]. Nevertheless, patients from the same family carrying the same *OPA1* variant show a variable phenotype strongly indicating that there are other environmental influences or genetic modifiers at play [54].

*OPA1* codes for an inner mitochondrial membrane fusion protein which has been shown to be critical in mitochondrial network formation, bioenergetics, mitophagy and mtDNA stability [55–57]. *OPA1* is ubiquitously expressed, but it is found at higher levels in metabolically active tissues such as neural tissue and cardiomyocytes. Why variants in a ubiquitously expressed gene only affect RGCs in the vast majority of cases is unknown but the observation that optic neuropathies are a common feature of mitochondrial disease suggests that RGCs are uniquely vulnerable to mitochondrial dysfunction. A potential explanation may include an inherent vulnerability due to their long axons, which are unmyelinated before the lamina cribrosa, and their relatively high bioenergetic demands, leading to a structural/metabolic chokepoint in the prelaminar segment [58, 59].

#### Optic neuropathies—management

The most critical step in managing optic neuropathies is establishing the diagnosis and confirming the causative genetic mutation. Although IONs have a classical disease presentation, this sometimes only becomes evident in retrospect. In the acute setting, particularly for patients with LHON who can initially present with unilateral vision loss, investigations are needed to rule out other causes of a rapidly progressive, severe optic neuropathy. When there is a high degree of clinical suspicion, patients can be genetically screened with a targeted gene panel in the first instance and, if negative, further testing such as whole exome or genome sequencing can be considered depending on local availability.

Establishing the underlying genetic defect is critical as it allows a better prediction of visual outcomes, may prompt screening for extraocular features (e.g. metabolic evaluation in Wolfram syndrome), and facilitates reproductive counselling. Given the variable phenotypes associated with the two commonest IONs, DOA and LHON, establishing a genetic cause allows patients to make informed decisions based on their likely visual decline.

There are limited pharmacological treatments for the mitochondrial optic neuropathies [42]. Idebenone, a synthetic hydrosoluble analogue of CoQ10, is the only treatment approved by the European Medicines Agency for LHON. In the United Kingdom, idebenone is approved for reimbursement in Scotland, Northern Ireland and Wales, but not in England. The current body of evidence indicates that idebenone can improve or stabilise vision in individuals with LHON up to 5 years from onset [60]. Early initiation of treatment after onset of vision loss and treatment duration of at least 12 months appears to be associated with a greater likelihood of improving or stabilising vision [61]. The 2017 ‘International Consensus Statement on the Clinical and Therapeutic Management of Leber Hereditary Optic Neuropathy’ recommended that idebenone be started as soon as possible at 900 mg/day in patients with disease less than 1 year, and treatment continued for at least 1 year to assess response [62]. If a clinically relevant response was confirmed, treatment should be continued for another 1 year. Idebenone has also been trialled in the treatment for DOA and a retrospective case series indicated a possible benefit of idebenone in visual stabilisation/recovery [63]. A properly powered randomised clinical trial with a standardised assessment protocol is needed to confirm whether idebenone can truly alter the natural history of this mitochondrial optic neuropathy.

Substantial progress has been made in the development of gene therapy for LHON in the last decade by capitalising on the technique of allotopic expression [60]. The first human clinical trial of gene therapy, with allotopic expression of a nuclear-encoded version of the wild-type *MT-ND4* gene (for the m.11778G>A mutation causing LHON) was commenced in 2011 [64]. At least seven other clinical trials have been completed since 2011 [60], including three phase III randomised, double-masked, placebo- or sham-controlled clinical trials (RESCUE, REVERSE and REFLECT) [65–67]. Other mutation-specific treatments, including therapy for the m.3460G>A mutation in *MT-ND1*, are in pre-clinical development or early phase clinical trials [10]. However, mutation-specific therapies are unlikely to be available for all patients. Mutation-independent gene therapies that aim to improve mitochondrial respiration, reduce mitochondrial stress, inhibit or delay RGC apoptosis, and promote RGC survival are attractive as they can potentially be utilised in all patients with an ION in combination with other neuroprotective therapies, if available.

Technological innovations in gene therapy and gene editing techniques have facilitated the development of alternative approaches that do not rely on the technique of allotopic expression. One strategy that is currently under pre-clinical investigation involves the delivery of wild-type mtDNA using an adeno-associated viral vector (AAV) furnished with a mitochondrially-targeting sequence; [68] mitochondrial gene editing using mitochondrially targeted zinc finger nucleases and transcription activator-like effector nucleases to induce heteroplasmy shift; [69] and mitochondrial base-pair editing using a cytosine base editor to correct point mutations in mtDNA causing mitochondrial disease [70]. Another approach is the use of mitochondrial replacement therapy (MRT) or mitochondrial donation to reduce transmission of mtDNA mutation from a female carrier to their offspring [71]. This novel in vitro fertilisation technique, approved in the United Kingdom in 2015, involves removing nDNA from an oocyte/zygote from a female carrier of a pathogenic mtDNA mutation and transferring the nDNA to a healthy donor oocyte/zygote [72]. The resulting reconstructed oocyte/zygote contains predominantly wild-type mtDNA from a donor; thereby reducing the risk of transmitting mitochondrial disease to the offspring of female carriers of mtDNA mutations.

There are several genetic therapies which are under development for DOA caused by *OPA1* mutations. One of the challenges in designing a genetic therapy for DOA is the size of the gene (>90 kb) making it too large for traditional vectors such as AAV. As a result, most current attempts include the use of modifying gene expression through molecules such as antisense oligonucleotides, ASOs. Strategies under pre-clinical investigation include an ASO that downregulates non-functional/mutant transcript and thus increases *OPA1* expression (Stoke Therapeutics, Massachusetts, USA); [73] and a related peptide-conjugated phosphorodiamidate morpholino oligomer molecule that also increases *OPA1* expression (PYC Therapeutics, Perth, Australia) [74]. Theoretically, transcriptional modifiers have the advantage over gene editing in not being constrained by the patient variant and there are less concerns about a potentially deleterious supraphysiological expression of *OPA1*. Work is ongoing to determine the extent to which ASOs are improving *OPA1* transcript levels and what increase in *OPA1* expression is needed to result in a clinically meaningful attenuation of RGC loss.

## Retrochiasmal vision loss

Some patients with mitochondrial disease can experience visual symptoms due to lesions involving the retrochiasmal visual pathways. This is most commonly seen with the m.3243A>G mutation in *MT-TL1*, which accounts for ~80% of cases of MELAS, and is also associated with MIDD [75]. Clinical features of MELAS include stroke-like episodes <40 years; seizures and/or dementia; and muscle weakness with ragged-red fibres on muscle biopsy and/or lactic acidaemia [76]. Stroke-like episodes may be precipitated by acute illness, surgery, and medications (including sodium valproate), leading to cerebral dysfunction accompanied by migraine-like prodromes, acute confusional states and focal seizures. Posterior brain regions are typically involved in the stroke-like episodes, potentially leading to homonymous visual field defects and detectable thinning of the ganglion cell complex layer on OCT, due to trans-synaptic retrograde degeneration of the RGC [77]. Other reported visual symptoms include cortical blindness, visual anosognosia (Anton syndrome), prosopagnosia, and regional geographic disorientation. Patients with MELAS are also prone to developing pigmentary retinopathy, with the risk of RPE degeneration proportional to the overall disease severity and mutant load [78].

### Retrochiasmal vision loss—management

Managing the retrochiasmal manifestations of mitochondrial diseases is challenging. There are currently no licensed disease-modifying treatments. Medications that have been trialled to prevent or treat stroke-like episodes in MELAS include coenzyme-Q10, L-arginine, L-carnitine and high-dose taurine supplementation [79]. Treatment for MELAS is generally supportive and may include referral to low vision service for those with visual impairment; cochlear implantation for those with sensorineural hearing loss; anticonvulsant therapy for seizure prevention (avoiding sodium valproate), and screening for other organ involvement including cardiac conduction defects, diabetes, kidney disease, and other ocular manifestations.

## Conclusion

Due to their predilection for metabolically active tissues, the eye is involved in over half of all patients with mitochondrial disease; affecting the extraocular muscles, retina and optic nerves. Although often considered as rare diseases with little or no effective treatments, significant progress has been made in the development of gene therapy and gene editing platforms, in particular for patients with mitochondrial optic neuropathies. A paradigm shift in therapy for mitochondrial disorders is underway and this must be reflected in a dramatic alternation in our clinical management of these patients: early and accurate molecular diagnosis must be the standard of care for these patients. In order to have access to nascent gene therapies and the exciting range of therapeutic trials underway in this traditionally underserved area of ophthalmology, patients need timely, expert neuro-ophthalmological evaluation and genetic testing.

## Summary

- Ophthalmic manifestations of mitochondrial disease include, but are not limited to, progressive external ophthalmoplegia, retinopathy, optic neuropathy, and deficiencies of the retrochiasmal visual pathways.
- Although traditionally recognised by ‘classic’ presentations, genotype–phenotype correlations in mitochondrial disease are imprecise; all patients should have genetic testing even if the presentation is not ‘classic’.
- Recent advancements in the development of gene therapy and gene editing techniques for mitochondrial diseases underscore the importance of timely neuro-ophthalmological evaluation and genetic testing.
